# Effect of Ca^2+^ Efflux Pathway Distribution and Exogenous Ca^2+^ Buffers on Intracellular Ca^2+^ Dynamics in the Rat Ventricular Myocyte: A Simulation Study

**DOI:** 10.1155/2014/920208

**Published:** 2014-05-29

**Authors:** Michal Pásek, Jiří Šimurda, Clive H. Orchard

**Affiliations:** ^1^Institute of Thermomechanics, Branch Brno, Academy of Science of the Czech Republic, Technická 2, 61669 Brno, Czech Republic; ^2^Department of Physiology, Faculty of Medicine, Masaryk University, Kamenice 5, 62500 Brno, Czech Republic; ^3^School of Physiology and Pharmacology, University of Bristol, Bristol BS8 1TD, UK

## Abstract

We have used a previously published computer model of the rat cardiac ventricular myocyte to investigate the effect of changing the distribution of Ca^2+^ efflux pathways (SERCA, Na^+^/Ca^2+^ exchange, and sarcolemmal Ca^2+^ ATPase) between the dyad and bulk cytoplasm and the effect of adding exogenous Ca^2+^ buffers (BAPTA or EGTA), which are used experimentally to differentially buffer Ca^2+^ in the dyad and bulk cytoplasm, on cellular Ca^2+^ cycling. Increasing the dyadic fraction of a particular Ca^2+^ efflux pathway increases the amount of Ca^2+^ removed by that pathway, with corresponding changes in Ca^2+^ efflux from the bulk cytoplasm. The magnitude of these effects varies with the proportion of the total Ca^2+^ removed from the cytoplasm by that pathway. Differences in the response to EGTA and BAPTA, including changes in Ca^2+^-dependent inactivation of the L-type Ca^2+^ current, resulted from the buffers acting as slow and fast “shuttles,” respectively, removing Ca^2+^ from the dyadic space. The data suggest that complex changes in dyadic Ca^2+^ and cellular Ca^2+^ cycling occur as a result of changes in the location of Ca^2+^ removal pathways or the presence of exogenous Ca^2+^ buffers, although changing the distribution of Ca^2+^ efflux pathways has relatively small effects on the systolic Ca^2+^ transient.

## 1. Introduction

During the last few years, it has become apparent that the ultrastructure of cardiac ventricular myocytes is critical to their function, with localised ion handling and signalling microdomains playing a key role in cell function. For example, Ca^2+^ influx via L-type Ca^2+^ current (*I*_Ca_) causes local Ca^2+^ release from adjacent sarcoplasmic reticulum (SR) at the dyad [1, 2]; and Ca^2+^ released from SR appears to have “privileged” access to the Na^+^/Ca^2+^ exchanger (NCX, [3]), presumably because of the proximity of NCX to SR Ca^2+^ release channels.

Ca^2+^ within the dyad—the site of Ca^2+^ entry via *I*_Ca_ and Ca^2+^ release from the SR—is critical, because it controls Ca^2+^-induced Ca^2+^ release (CICR) from the SR [1, 2] and Ca^2+^-dependent inactivation (CDI) of *I*_Ca_ [4]. Similarly, bulk cytoplasmic Ca^2+^ is critical since it determines contraction and relaxation. Colocation of different Ca^2+^ flux pathways is also likely to be important in Ca^2+^ “autoregulation” [5], whereby an increase in intracellular Ca^2+^ increases efflux via NCX and decreases influx via *I*_Ca_ [6], and in the genesis of some types of arrhythmia (e.g., delayed afterdepolarizations), which are caused by activation of inward NCX current by spontaneous SR Ca^2+^ release [7, 8]. Such localisation may also change in pathological conditions, thereby altering cell function.

Many studies have investigated the location and colocation of Ca^2+^ influx and release pathways and their importance for cell function. Although Ca^2+^ efflux occurs predominantly in the t-tubules, which are, therefore, likely to play a role in ensuring rapid and uniform relaxation of the cell [9, 10], less is known about the ultrastructural location and colocation of Ca^2+^ efflux pathways and how critical such location is to cell function. While it appears likely that the localisation of NCX is important in cell function (above), little is known about the relevance of the distribution of SR Ca^2+^ ATPase (SERCA), which biochemical studies have shown throughout the SR [11], while immunohistochemical studies suggest that it is located predominantly at the Z-line and, thus, close to the t-tubules and the site of SR Ca^2+^ release [12]. We have, therefore, used a computer model of the rat ventricular myocyte to explore the sensitivity of intracellular Ca^2+^ cycling to changes in dyadic Ca^2+^ handling brought about either by altering the distribution of Ca^2+^ efflux pathways between the dyad and bulk cytoplasm or by addition of Ca^2+^ buffers that are used experimentally to differentially buffer Ca^2+^ within the dyad and bulk cytoplasm.

## 2. Methods

**Figure 1:**
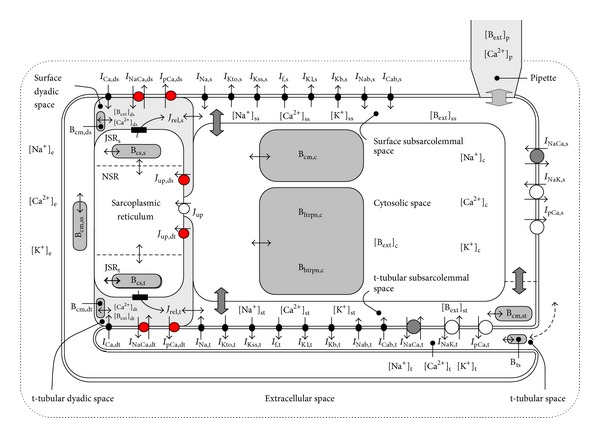
Schematic diagram of the rat ventricular cell compartmental model used in the present study. The description of the electrical activity of surface membrane (s, ds at surface dyads) and t-tubular membrane (t, dt at t-tubular dyads) comprises formulations of the following ion currents: fast sodium current (*I*_Na_), L-type calcium current (*I*_Ca_), transient outward potassium current (*I*_Kto_), steady-state outward potassium current (*I*_Kss_), inward rectifying potassium current (*I*_K1_), hyperpolarization-activated current (*I*_f_), background currents (*I*_Kb_, *I*_Nab_, and *I*_Cab_), sodium-calcium exchange current (*I*_NaCa_), sodium-potassium pump current (*I*_NaK_), and calcium pump current (*I*_pCa_). The intracellular space contains the cytosolic space (c), surface and t-tubular subsarcolemmal spaces (ss, st), surface and t-tubular dyadic spaces (ds, dt), network and junctional compartments of sarcoplasmic reticulum (NSR, JSR_s_, and JSR_t_), endogenous Ca^2+^ buffers (calmodulin (B_cm_), troponin (B_htrpn_, B_ltrpn_), and calsequestrin (B_cs_)), and exogenous Ca^2+^ buffer (e.g., BAPTA or EGTA (B_ext_)). B_ts_ denotes the nonspecific Ca^2+^ buffer associated with luminal part of t-tubular membrane. *J*_up_ represents Ca^2+^ flow via SR Ca^2+^ pump and the small filled rectangles in JSR membrane ryanodine receptors. The small bidirectional arrows denote Ca^2+^ diffusion. Ion diffusion between the t-tubular and extracellular spaces is represented by the dashed arrow. The changes with respect to the previous model [13] are highlighted in red. The Matlab code of the model can be downloaded at: http://www.it.cas.cz/en/d3/l033/biophysics-cardiac-cells.

The model used in this study (Figure 1) was based on that described by Pásek et al. [13], which was modified to explore the effect of the distribution of Ca^2+^ removal pathways on intracellular Ca^2+^ dynamics. The distribution of NCX, sarcolemmal Ca^2+^ ATPase, and L-type Ca^2+^ channels between the t-tubular and surface membranes was as determined experimentally and described in [13]. The fraction of the Ca^2+^ extrusion pathways located at the t-tubular and surface membrane dyads (*f*_NaCa,d_ and *f*_pCa,d_) was varied independently between 0 (their normal value in the model) and 0.3, with reciprocal variation of the corresponding extradyadic fraction at each membrane. Thus, when *f*_NaCa,d_ or *f*_pCa,d_ was set to 0.3, the fractions of the corresponding ion transporter at t-tubular dyadic space, surface dyadic space, t-tubular subsarcolemmal space, and surface subsarcolemmal space were, respectively, 0.3 × t-tubular fraction of ion transporter, 0.3 × surface fraction of ion transporter, 0.7 × t-tubular fraction of ion transporter, and 0.7 × surface fraction of ion transporter. The fraction of L-type Ca^2+^ channels located at the dyads (*f*_Ca,d_) was maintained constant at 1.

The fraction of SR Ca^2+^ ATPase (SERCA) at the dyad (*f*_up,d_) was also varied between 0 (its normal value in the model) and 0.3, with the remaining fraction 1 − *f*_up,d_ in the bulk cytosolic space. The distribution of the dyadic fraction of SERCA between the surface and t-tubular dyads was set proportional to the fraction of dyads at the t-tubular and surface membranes (0.8 and 0.2, resp. [13]). Thus, for a total dyadic fraction of SERCA of 0.3, its fraction at the t-tubular membrane was 0.3 × 0.8 and that at surface membrane was 0.3 × 0.2. To simplify presentation, unless stated otherwise (e.g., Figure 7), data are shown only for t-tubular dyads, since there are more dyads at the t-tubules than at the surface membrane [14], so that they play a greater role in excitation-contraction coupling, even though the gain of CICR appears to be similar at the two sites [15]. The behaviour of dyads at the two sites in response to changes in the distribution of Ca^2+^ efflux pathways was also qualitatively similar in the present study.

Extracellular [Ca^2+^], [Na^+^], and [K^+^] were normally set to 1.2 mM, 140 mM, and 5.4 mM, respectively. To simulate the addition of EGTA and BAPTA to the intracellular compartments from a patch pipette, the diffusion of Ca^2+^ and exogenous Ca^2+^ buffers (with or without bound Ca^2+^) among the pipette, subsarcolemmal spaces, dyadic spaces, and cytosol was described using ordinary differential equations where the flux of Ca^2+^ and Ca^2+^ buffer between adjacent compartments is directly proportional to the concentration difference of Ca^2+^ or Ca^2+^ buffer between the compartments and inversely proportional to the time constant of Ca^2+^ or buffer exchange, as described previously [13]. To replicate the experimental conditions used to determine the effect of EGTA and BAPTA on *I*_Ca_ inactivation during voltage clamp, trains of six voltage pulses (200 ms, 0.1 Hz) from −80 to 0 mV were used, and inactivation during the 6th pulse was analyzed [15]. The following ion concentrations were used for the extracellular compartment, in the pipette, and intracellular compartment: [Ca^2+^]_e_ = 1 mM; [Na^+^]_e_ and [K^+^]_e_≈ 0 (10^3^ × lower than normal, that is, 0.135 and 0.005 mM, resp.); [Ca^2+^]_p_ = 0.5 nM; [Na^+^]_i_ and [K^+^]_i_≈ 0 (0.01 and 0.14 mM, resp.), reflecting their dialysis via the pipette. K^+^ currents were disabled because they were blocked experimentally using Cs^+^ [15].

The simulations investigating the effect of Ca^2+^ efflux pathway distribution on intracellular Ca^2+^ dynamics under current clamp conditions were performed at 5 Hz steady-state stimulation, which corresponds to resting heart rate in the rat. The simulations investigating the effect of EGTA and BAPTA under current clamp were performed at 1 Hz steady-state stimulation, because Ca^2+^ overload was observed in the model cell at higher stimulation rates in the presence of these buffers.

Numerical computation of the system of 97 nonlinear differential equations was performed in MATLAB v.7.2 (MathWorks, Natick, MA, USA) using the solver for stiff system ODE-15s. The model equations were simultaneously solved using a time-step adjusted to keep the estimated relative error of inner variables below a threshold value of 10^−6^. To attain dynamic steady state in the model cell at both stimulation frequencies (1 Hz and 5 Hz), the model was paced for 600 s of equivalent cell life time. The basic units in which the equations were solved were the following: mV for membrane potential, mA for membrane currents, mM for ionic concentrations, s for time, and mL for volumes.

## 3. Results

### 3.1. The Effect of Ca^2+^ Removal Pathway Distribution on Ca^2+^ Dynamics

**Figure 2:**
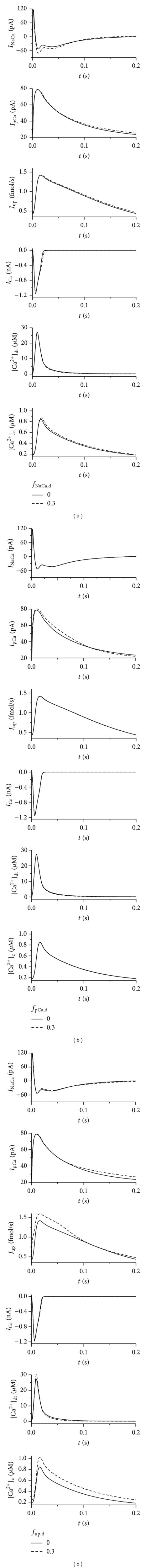
Effect of partial localization of NCX exchange (a), sarcolemmal Ca^2+^ pump (b), and SERCA (c) at the dyads on the time course of total *I*_NaCa_, *I*_pCa_, *J*_up_, and *I*_Ca_ and Ca^2+^ transients in the t-tubular dyadic space ([Ca^2+^]_dt_) and in the cytosol ([Ca^2+^]_c_) during a steady-state cycle at 5 Hz (action potentials elicited by 1 ms current clamps). The solid and dashed lines, respectively, represent traces obtained in control conditions (*f*_NaCa,d_ = *f*_pCa,d_ = *f*_up,d_ = 0) and when*f*_NaCa,d_, *f*_pCa,d_, or *f*_up,d_ was separately increased to 0.3.

**Figure 3:**
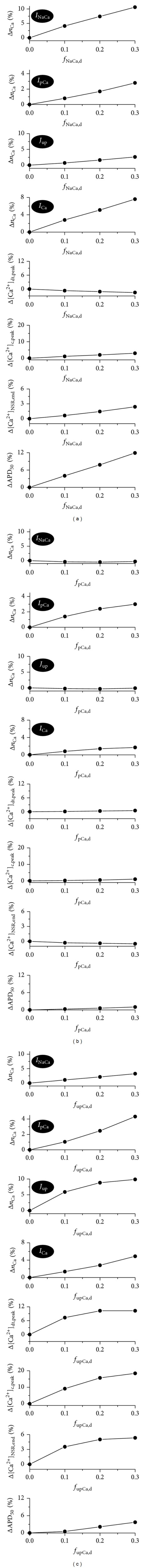
Effect of partial localization of NCX exchange (a), sarcolemmal Ca^2+^ pump (b), and SERCA (c) at the dyads on the amount of Ca^2+^ (*n*_Ca_) transferred through *I*_NaCa_, *I*_pCa_, *J*_up_, and *I*_Ca_; the peak value of Ca^2+^ transients in the t-tubular dyadic space ([Ca^2+^]_dt,peak_) and in the cytosol ([Ca^2+^]_c,peak_); the end-diastolic level of Ca^2+^ concentration in the network SR ([Ca^2+^]_NSR,end_); and action potential duration at 50% repolarization (APD_50_) during a steady-state cycle at 5 Hz. The fraction of all three transporters at the dyads was increased separately from control (*f*_NaCa,d_ = *f*_pCa,d_ = *f*_up,d_ = 0) to 0.1, 0.2, and 0.3. All values are expressed as a percentage relative to those obtained in control conditions.

Figure 2 shows the effect of increasing the dyadic fraction of NCX (a), sarcolemmal Ca^2+^ pump (b), and SERCA (c), from 0 to 0.3, on whole cell transmembrane Ca^2+^ fluxes and dyadic and cytoplasmic Ca^2+^ transients during a steady-state beat at 5 Hz. Increasing dyadic NCX resulted in increased Ca^2+^ efflux via NCX, reflected as an increase in peak inward *I*_NaCa_, as expected from exposure of 30% of NCX to the high Ca^2+^ in the dyadic space. This was accompanied by a small increase in the amplitude of the cytoplasmic Ca^2+^ transient, partly as a result of reduced Ca^2+^ removal via NCX from the bulk cytoplasm. The increase of cytosolic Ca^2+^ and decrease of cytosolic NCX resulted in a small increase in Ca^2+^ removal via the sarcolemmal Ca^2+^ ATPase and SERCA (see *I*_pCa_ and *J*_up_, resp.). The small prolongation of *I*_Ca_ is mainly the result of APD prolongation (see Figure 3), due primarily to the increase of *I*_NaCa_, rather than to decreased CDI. Increasing the dyadic sarcolemmal Ca^2+^ pump fraction resulted in an increase in Ca^2+^ efflux via the pump but had little effect on the other Ca^2+^ fluxes or concentrations, reflecting its relatively minor role in Ca^2+^ handling. In contrast, increasing dyadic SERCA resulted in increased SR Ca^2+^ uptake that was accompanied by an increase in the amplitude of the dyadic and cytoplasmic Ca^2+^ transients as a result of increased SR Ca^2+^ release, and thus a small increase in Ca^2+^ extrusion via NCX and the sarcolemmal Ca^2+^ pump during the later stages of the Ca^2+^ transient.

The dyadic Ca^2+^ transient depends not only on Ca^2+^ entry into the dyad via *I*_Ca_ and SR Ca^2+^ release and the absence or presence of Ca^2+^ extrusion pathways at the dyad but also on Ca^2+^ diffusion from the dyad into adjacent cytoplasm. In the absence of Ca^2+^ extrusion pathways at the dyad, all of the Ca^2+^ leaves the dyad by diffusion, reaching a maximum value of Ca^2+^ flux from t-tubular dyadic space to t-tubular subsarcolemmal space of 11.23 fM/s at the peak of the dyadic Ca^2+^ transient. Increasing the fraction of NCX or sarcolemmal Ca^2+^ ATPase at the dyad to 0.3 had little effect on this flux, while increasing the fraction of SERCA at the dyad to 0.3 increased its maximum value to 12.4 fM/s, as a result of the increase in the amplitude of the dyadic Ca^2+^ transient. The fraction of dyadic [Ca^2+^] leaving the dyad by diffusion decreased from 1 under control conditions to 0.81 when the fraction of SERCA at the dyad was increased to 0.3.

Figure 3 shows the percentage changes in integrated steady-state Ca^2+^ fluxes, Ca^2+^ concentrations, and action potential duration as the dyadic fraction of NCX (a), sarcolemmal Ca^2+^ pump (b), and SERCA (c) was increased in 0.1 steps from 0 to 0.3. This shows gradation of the changes described above with increasing dyadic fractions of these Ca^2+^ removal pathways. It is worth noting that increasing dyadic NCX, in addition to the changes described above, led to a small increase in SR Ca^2+^ content, as a result of increased Ca^2+^ uptake via SERCA as a consequence of a larger cytosolic Ca^2+^ transient induced partly by lower extradyadic fraction of NCX. Changing the dyadic fraction of the sarcolemmal Ca^2+^ pump had little effect on other aspects of Ca^2+^ handling, as noted above. Figure 3 also shows the increase in SR Ca^2+^ content that resulted from the increase in dyadic SERCA and shows increased Ca^2+^ influx via *I*_Ca_ as a result of reduced CDI due to more rapid Ca^2+^ removal from the dyad by SERCA and a slight increase in action potential duration (APD) as a result of the changes in *I*_Ca_ and *I*_NaCa_.

We also explored the effect of simultaneously increasing the dyadic fraction of the three Ca^2+^ removal pathways to 0.3 to investigate the effect of competition between the extrusion pathways at the dyad (not shown). This increased Ca^2+^ removal by NCX, SL Ca^2+^ ATPase, and SERCA by 10.3%, 8.9%, and 13.7%, respectively, and increased the Ca^2+^ load of the cell. End-diastolic [Ca^2+^] in the network SR (NSR) increased by 9.13%, while that in the cytoplasm increased by 45.6% to 275 nM; the peak of the dyadic Ca^2+^ transient at the t-tubules increased by 9.9%, while the peak of the cytoplasmic Ca^2+^ transient increased by 30.2%. Action potential duration at 50% repolarization (APD_50_) increased by 16%, and Ca^2+^ influx via *I*_Ca_ increased by 11.8%. These data highlight the interaction between the different Ca^2+^ efflux pathways and their location, showing that changes in the location of a single pathway can alter the activity of the other Ca^2+^ flux pathways, as well as Ca^2+^ concentrations in subcellular spaces and the electrophysiology of the cell.

### 3.2. The Effect of Buffering Ca^2+^ in Different Compartments on Ca^2+^ Dynamics

EGTA and BAPTA have been widely used experimentally to buffer bulk cytoplasmic Ca^2+^ and cytoplasmic plus dyadic Ca^2+^, respectively. Given the effects of changing the distribution of Ca^2+^ efflux pathways on Ca^2+^ dynamics described above, we have investigated the effects of these buffers on Ca^2+^ dynamics in the model (at *f*_NaCa,d_ = *f*_pCa,d_ = *f*_up,d_ = 0 and *f*_Ca,d_ = 1).

**Figure 4:**
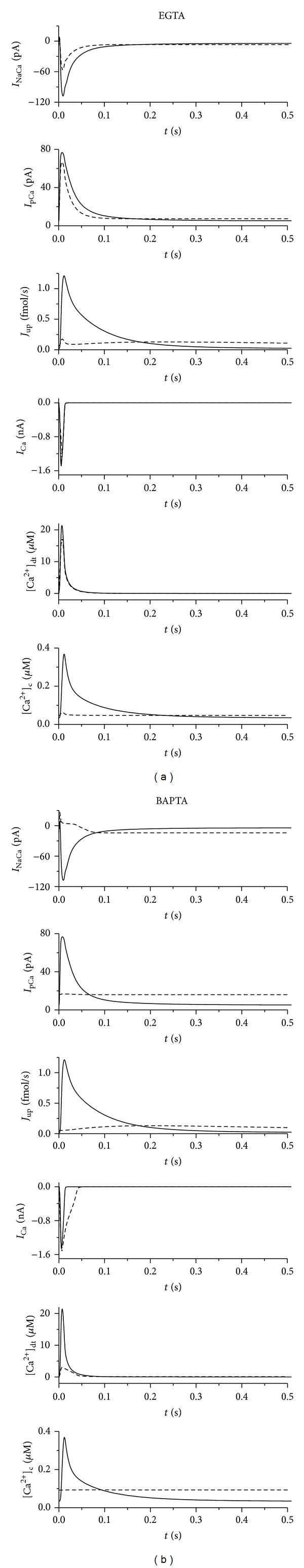
Effect of 10 mM EGTA (a) and 10 mM BAPTA (b) on the time course of total *I*_NaCa_, *I*_pCa_, *J*_up_, and *I*_Ca_ and Ca^2+^ transients in the t-tubular dyadic space ([Ca^2+^]_dt_) and in the cytosol ([Ca^2+^]_c_) during the first 0.5 s of steady-state cycle at 1 Hz (action potentials elicited by 1 ms current clamps). The solid and dashed lines show, respectively, the traces obtained in control conditions and in the presence of the buffers in the intracellular space.

Figure 4 shows the effect of 10 mM EGTA (a) or BAPTA (b) on the same Ca^2+^ fluxes and concentrations shown in Figure 2. In support of the idea that these buffers differentially affect [Ca^2+^] in the dyadic and cytoplasmic spaces, EGTA decreased the amplitude of the dyadic Ca^2+^ transient by ~17%, but it almost completely abolished the cytoplasmic Ca^2+^ transient, decreasing its amplitude by >90%. In contrast, BAPTA decreased the dyadic Ca^2+^ transient by ~86% and completely inhibited the cytosolic Ca^2+^ transient. As a result, these buffers had different effects on Ca^2+^ fluxes.

EGTA decreased Ca^2+^ extrusion via NCX during the initial 170 ms of the cycle as a result of the decreased Ca^2+^ transient in the dyadic and subsarcolemmal spaces. However, subsequent Ca^2+^ extrusion via NCX, until the end of the cycle, was higher than in the absence of EGTA, as a result of higher [Ca^2+^] in both spaces ([Ca^2+^]_dt,end_: 38 nM versus 26 nM in control, [Ca^2+^]_c,end_: 47 nM versus 34 nM in control) due to Ca^2+^ release from the buffer. Similar changes were observed for the SL Ca^2+^ pump and SERCA, although for these extrusion pathways the increase of Ca^2+^ extrusion during the later phase of the cycle is greater than its decrease during the Ca^2+^ transient. Similarly, the faster Ca^2+^ buffer BAPTA decreased Ca^2+^ extrusion via NCX during the initial phase of the cycle (80 ms). However, subsequent Ca^2+^ extrusion via NCX was substantially greater in the presence of BAPTA as a result of the higher [Ca^2+^] in both spaces ([Ca^2+^]_dt,end_: 95 nM versus 26 nM in control, [Ca^2+^]_c,end_: 92 nM versus 34 nM in control) due to Ca^2+^ released from the buffer. A similar change was observed for the SL Ca^2+^ pump. The different effect of EGTA and BAPTA on *I*_Ca_ reflects their different efficiency to reduce Ca^2+^ transient in the dyads and thus to suppress Ca^2+^-induced inactivation of *I*_Ca_.

**Figure 5:**
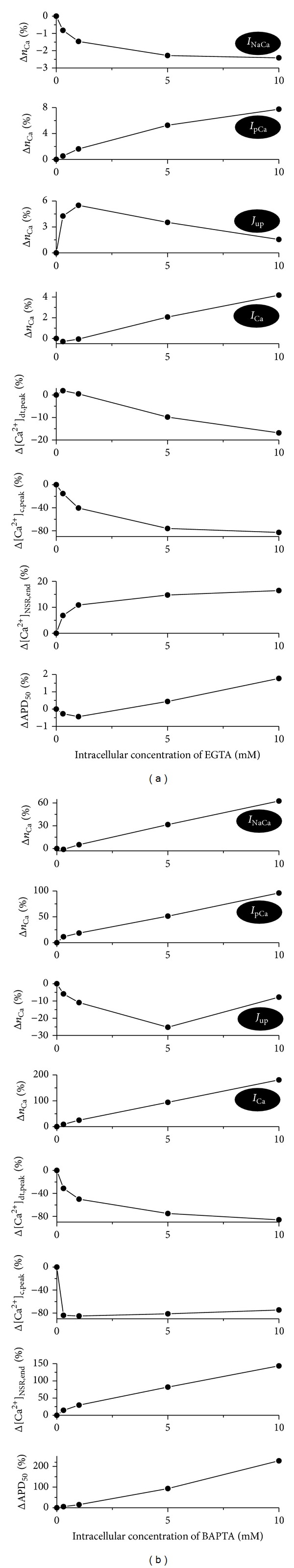
Effect of EGTA (a) and BAPTA (b) on the amount of Ca^2+^ (*n*_Ca_) transferred through *I*_NaCa_, *I*_pCa_, *J*_up_, and *I*_Ca_, the peak value of Ca^2+^ transients in the t-tubular dyadic space ([Ca^2+^]_dt,peak_) and in the cytosol ([Ca^2+^]_c,peak_), the end-diastolic level of Ca^2+^ concentration in NSR ([Ca^2+^]_NSR,end_), and APD_50_ during a steady-state cycle at 1 Hz. All values are expressed as a percentage relative to those obtained in control conditions. Positive values of Δ*n*_Ca_ related to *I*_NaCa_, *I*_pCa_, and *J*_up_ indicate an increase of Ca^2+^ extrusion while positive values related to *I*_Ca_ indicate increased Ca^2+^ entry. Buffer concentrations were 0.3, 1, 5, and 10 mM.

The concentration dependence of the effects of EGTA and BAPTA is illustrated in Figure 5, which shows the percentage changes in the amount of Ca^2+^ transferred by each efflux pathway (calculated as the integral of the corresponding flux during a steady-state cycle at 1 Hz), Ca^2+^ concentrations in intracellular spaces, and AP duration. Over the concentration range investigated, Ca^2+^ extrusion via SERCA is increased by EGTA and decreased by BAPTA, while the opposite holds for extrusion via NCX. The complex relationship between [buffer] and Ca^2+^ flux via SERCA is due to the dynamics of [Ca^2+^] changes in the dyads and the cytosol during the two phases described for Figure 4, which are different for BAPTA and EGTA. In general terms, Ca^2+^ flux via SERCA is reduced during the Ca^2+^ transient, since Ca^2+^ transient amplitude is reduced by the buffer; following the Ca^2+^ transient, Ca^2+^ flux via SERCA is enhanced as Ca^2+^ is released from the buffer; this results in an increase of [Ca^2+^]_NSR,end_ (by 16% for EGTA and 143% for BAPTA). Figure 5 also shows that, while 10 mM EGTA increases Ca^2+^ influx via *I*_Ca_ and the associated APD_50_ slightly (4.2% and 1.8%, resp.), BAPTA at the same concentration affects both profoundly (181% and 227%, resp.) as a result of increased Ca^2+^ entry via *I*_Ca_ due to a marked decrease in CDI as a result of the smaller dyadic Ca^2+^ transient.

These data are consistent with the idea that EGTA and BAPTA have differential effects on Ca^2+^ buffering in the dyad and bulk cytoplasm and, thus, on CDI of *I*_Ca_, as suggested from the experimental data. We, therefore, investigated in more detail whether the model replicates the effect of these buffers on *I*_Ca_ and the mechanism of Ca^2+^ buffering.

**Figure 6:**
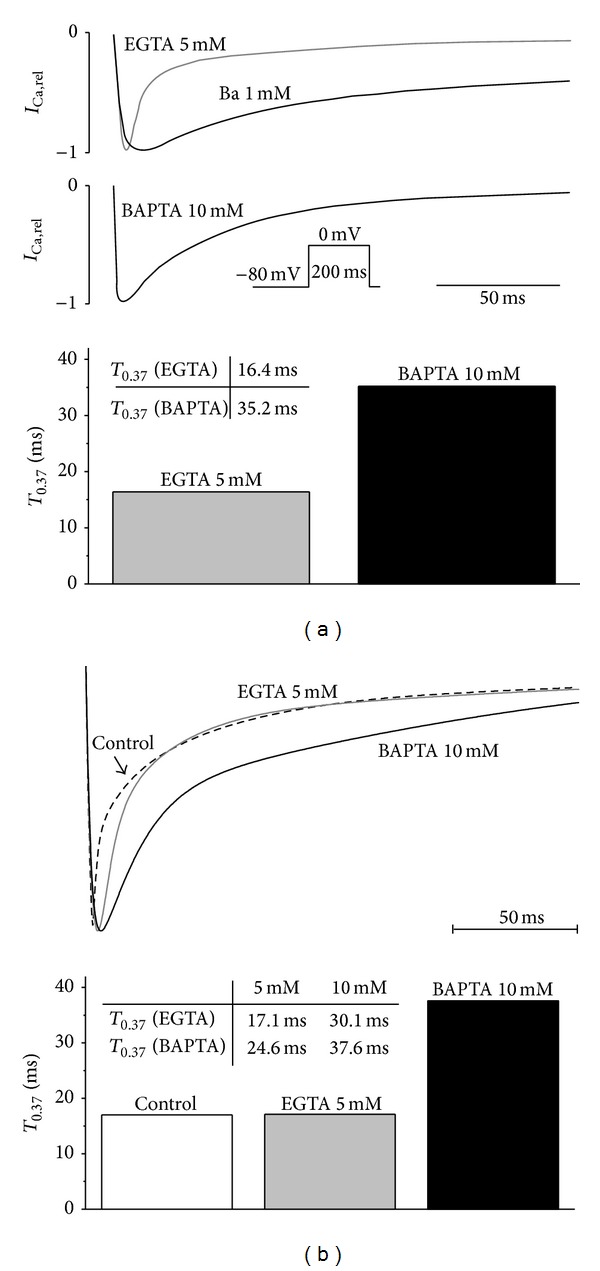
(a) Inactivation of *I*_Ca_ in the presence of intracellular EGTA (5 mM) or BAPTA (10 mM) in rat ventricular myocytes (adopted from [15]); *I*_Ca_ was recorded during a 200 ms depolarising pulse from −80 mV to 0 mV at room temperature. The kinetics of *I*_Ca_ inactivation were characterized by the time required for the current to decay to 0.37 of its peak amplitude (*T*_0.37_). The lower panel shows that *I*_Ca_ inactivation was substantially slowed in the presence of 10 mM BAPTA: *T*_0.37_ = 35.2 ms versus 16.4 ms in the presence of 5 mM EGTA. 1 mM Ba was used to show the time course of *I*_Ca_ in the absence of Ca^2+^-dependent inactivation. (b) Reconstruction of experimentally observed effect of EGTA and BAPTA on *I*_Ca_ inactivation in the model. The top panel shows superimposed normalized responses of *I*_Ca_ in control conditions and with 5 mM EGTA and 10 mM BAPTA. The lower panel shows corresponding values of *T*_0.37_.

Figure 6 shows the effect of EGTA (used experimentally to buffer bulk cytoplasmic Ca^2+^) and BAPTA (used to buffer cytoplasmic and dyadic Ca^2+^) on *I*_Ca_ in the model, compared with the experimental data.  Figure 6(a) shows experimental records of *I*_Ca_ in the presence of EGTA and BAPTA (top, 1 mM Ba was used to determine the time course of *I*_Ca_ in the absence of CDI) and the time required for the current to decay to 0.37 of the peak amplitude during inactivation (below). Corresponding records and data from the model obtained under the same conditions (see Section 2) are shown in Figure 6(b), showing that the model accurately replicates the experimental data.

**Figure 7:**
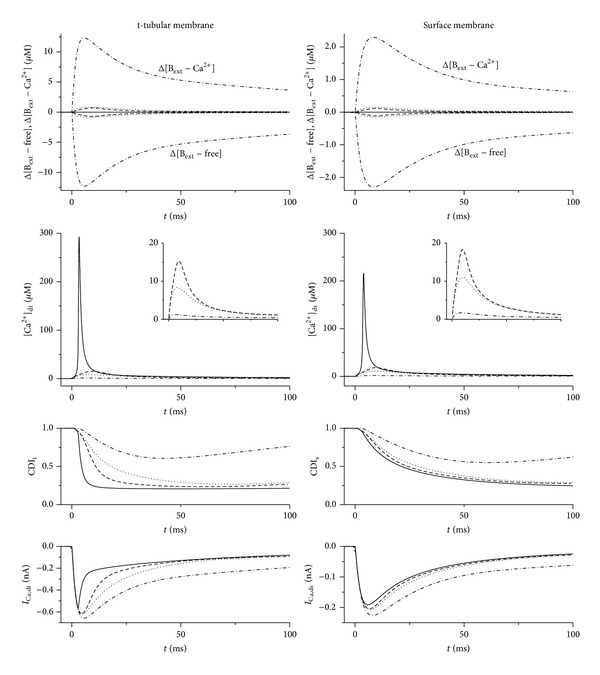
Effect of EGTA and BAPTA on intracellular Ca^2+^ and *I*_Ca_ inactivation at the t-tubular and surface membranes during a voltage clamp pulse from −80 to 0 mV in the model. The upper panels show the differences between concentrations in the dyadic and subsarcolemmal spaces of the buffers with bound Ca^2+^  (Δ[B_ext_ − Ca^2+^] = [B_ext_−Ca^2+^]_d_ − [B_ext_−Ca^2+^]_s_) and unbound Ca^2+^  (Δ[B_ext_ − free] = [B_ext_−free]_d_ − [B_ext_−free]_s_). The other panels show Ca^2+^ transients in both dyadic spaces ([Ca^2+^]_dt_, [Ca^2+^]_ds_), Ca^2+^-dependent inactivation of t-tubular and surface membranes *I*_Ca_ (CDI_t_, CDI_s_), and time courses of related current components (*I*_Ca,dt_ and *I*_Ca,ds_). The solid, dashed, dotted, and dashed-dotted lines show the data obtained in control conditions and in the presence of 5 mM EGTA, 10 mM EGTA, and 10 mM BAPTA, respectively. The insets in the graphs of [Ca^2+^]_dt_ and [Ca^2+^]_ds_ show the dyadic Ca^2+^ transients in the presence of Ca^2+^ buffers at a higher gain.

To understand these changes in more detail, we investigated the differences in concentration of Ca^2+^-bound and Ca^2+^-free buffer between the dyadic and adjacent subsarcolemmal spaces (Δ[B_ext_ − Ca^2+^] and Δ[B_ext_ − free] in the upper panels of Figure 7). The simulations revealed that the rates of buffer exchange (both Ca^2+^-bound and Ca^2+^-free) between the dyadic and adjacent subsarcolemmal spaces are critical to the efficacy of Ca^2+^ buffers in the dyadic space. Although both buffers inhibited the cytosolic Ca^2+^ transient (Figure 4), only BAPTA inhibited effectively the rise of Ca^2+^ in the dyadic space, thus causing significant inhibition of CDI of *I*_Ca_. The principal reason for the different potencies of BAPTA and EGTA in reducing the dyadic Ca^2+^ transient was their different rates of Ca^2+^ binding (EGTA: *k*_*on*⁡_ = 5000 mM^−1^ s^−1^; BAPTA: *k*_*on*⁡_ = 500000 mM^−1^ s^−1^). As a consequence, the concentration gradients of free BAPTA and Ca^2+^-BAPTA and free EGTA and Ca^2+^-EGTA between the dyadic space and bulk cytosol were substantially different after the onset of depolarisation. In the case of BAPTA, fast onset of large gradients provided large driving forces, causing rapid movement of Ca^2+^-BAPTA molecules out of the dyadic space and of free BAPTA into the dyadic space, so that BAPTA appeared to act as a fast “shuttle.” The slower rate of Ca^2+^ binding to EGTA and the consequent development of substantially smaller free EGTA and Ca^2+^-EGTA gradients between the dyadic space and cytosol reduced the ability of EGTA to affect dyadic [Ca^2+^] and, thus, Ca^2+^-induced inactivation of *I*_Ca_. The corresponding [Ca^2+^] in the dyads and its effect on CDI of *I*_Ca_ are shown in the other panels of Figure 7.

Since experimental data suggest that dyadic function is different at the t-tubules and surface membrane, with more rapidly inactivating *I*_Ca_ at the t-tubules [15], we also used the model to explore the effect of these buffers at the two sites. Figure 7 shows that, in control conditions, the dyadic Ca^2+^ transient during a voltage clamp pulse is larger at the t-tubules than at the surface membrane (293 *μ*M versus 216.7 *μ*M). This is due to a faster activation of SR Ca^2+^ release at the t-tubules caused by a faster rise of Ca^2+^ in t-tubular dyads at the beginning of the pulse. The main reason is the different volumes of t-tubular and surface subsarcolemmal spaces in the model (fractional volumes: *fV*_s,t_ = 0.35 and *fV*_s,s_ = 0.65, resp.), which were set to be proportional to the area of each membrane that is nonjunctional: 52% of t-tubular membrane and 92.3% of surface membrane [13, 14]. Both buffers reduced the dyadic Ca^2+^ transient (Figure 7), but to a greater extent at the t-tubules, so that in the presence of buffer the Ca^2+^ transient was even smaller in the t-tubular dyads than in the surface dyads. This is a consequence of changes in *I*_Ca_ and, hence, CICR, mainly as a result of reduced t-tubular *I*_Ca_ due to Ca^2+^ depletion in the t-tubular lumen [13]. This Ca^2+^ depletion is enhanced in the presence of exogenous buffer because of slowed inactivation of t-tubular *I*_Ca_.

## 4. Discussion

The first part of the present study was designed to investigate the effect of Ca^2+^ efflux pathway distribution on the Ca^2+^ dynamics of the cardiac ventricular myocyte. The reason for such an approach is that relatively little is known about the distribution of these pathways or its functional significance. For example, biochemical studies suggest that SERCA is located throughout the SR [11]. However, immunohistochemical studies have shown it predominantly at the Z-line and, thus, close to the t-tubules and the site of SR Ca^2+^ release [12]. The functional significance of such a location is unclear; it would be expected to result in futile Ca^2+^ cycling, unless this is minimized by rapid Ca^2+^ diffusion away from the site of release. However, functional data shows that Ca^2+^ entry during the latter part of *I*_Ca_ can load the SR with Ca^2+^ that is released in the subsequent contraction [16, 17]; this suggests that Ca^2+^ entering the cell via *I*_Ca_, which occurs predominantly at the t-tubules, is easily accessible to SERCA. Similarly, it has been suggested that Ca^2+^ released from the SR has “privileged access” to NCX [3]. This is consistent with the mainly t-tubular location of NCX, which places it in close proximity to the majority of SR Ca^2+^ release channels, which are also found predominantly at the t-tubules. However, this might be expected to be disadvantageous by producing futile Ca^2+^ cycling and enhancing arrhythmias caused by spontaneous SR Ca^2+^ release, although it may also contribute to autoregulation (see Section 1).

The present study shows that location of a Ca^2+^ efflux pathway at the dyad results in increased Ca^2+^ uptake by that pathway, as a result of exposure to the high dyadic [Ca^2+^]. The quantitative impact of changes in distribution was SERCA > NCX > sarcolemmal Ca^2+^ ATPase, as expected from their relative importance in Ca^2+^ removal from the cell cytoplasm [18]. Interestingly, a reduction in Ca^2+^ removal from the cell cytoplasm by one pathway (as a result of its redistribution to the dyad) resulted in an increase in the cytoplasmic Ca^2+^ transient and an increase in Ca^2+^ removal by the other two pathways. These data illustrate, therefore, (a) the competition between Ca^2+^ efflux pathways in a particular cellular compartment and (b) changes in another compartment due to the redistribution of Ca^2+^ efflux by one pathway.

The data also suggest that changes in distribution may alter cell function, for example, by altering the amplitude of the cytoplasmic Ca^2+^ transient and, hence, contraction, as a result of changes in SR Ca^2+^ content and/or Ca^2+^ extrusion from the cytoplasm; changes in dyadic Ca^2+^ may also alter the amount of Ca^2+^ released in response to *I*_Ca_. An increase in SR Ca^2+^ content—as, for example, when NCX in the dyad was increased—would be expected to be proarrhythmic, particularly when coupled to the increased NCX adjacent to the site of Ca^2+^ release. However, what is perhaps most striking is the stability of the cell to the imposed changes: even large (30%) changes in the distribution of Ca^2+^ efflux pathways led to relatively small disturbances of cell Ca^2+^ dynamics. This may reflect diffusional redistribution of Ca^2+^ between cell compartments and the presence of multiple flux pathways, which may be advantageous and protective in conditions such as HF in which changes in protein expression, distribution, and activity may occur.

Given these data, it was of interest to investigate the effect of perturbing cell Ca^2+^ handling using a different method. We chose to investigate the effect of the exogenous Ca^2+^ buffers EGTA and BAPTA, since they are used experimentally to buffer Ca^2+^ in different compartments of the cell-the bulk cytoplasm and the cytoplasm and dyad, respectively. Although the precise mechanism is unclear, the observation that BAPTA, but not EGTA, inhibits CDI of *I*_Ca_ supports the idea that they buffer Ca^2+^ in different compartments [19, 20].

Addition of EGTA or BAPTA to the model cell resulted in changes of *I*_Ca_ that were very similar to those observed experimentally [15]. In addition to providing credibility for the model, this also provides support for the use of these buffers in experiments, as described above. More interestingly, however, the model enabled us to investigate the mechanism of action of these buffers and suggested that, rather than acting as static buffers, they acted as shuttles. Thus, rapid binding of Ca^2+^ to BAPTA resulted in large concentration gradients, which caused rapid movement of Ca^2+^-BAPTA molecules out of the dyadic space and of free BAPTA into the dyadic space, so that BAPTA appeared to act as a fast “shuttle.” The slower rate of Ca^2+^ binding to EGTA and the consequent development of substantially smaller free EGTA and Ca^2+^-EGTA gradients between the dyadic space and cytosol reduced the ability of EGTA to affect dyadic [Ca^2+^] and, thus, CDI of *I*_Ca_.

The present work also suggests that EGTA and BAPTA have different effects on CDI of *I*_Ca_ at the t-tubular and surface membrane dyads. The effect of both buffers on CDI and, thus, on *I*_Ca_, CICR, and the Ca^2+^ transient is greater at the t-tubular dyad than at the surface dyad (Figure 7), because of the more marked CDI at the t-tubules under control conditions. However, in the presence of EGTA, CDI remains greater at the t-tubular dyad than at the surface membrane, while it is similar at the two sites in the presence of BAPTA.

It has been suggested that the more marked CDI of *I*_Ca_ at the t-tubules results in t-tubular *I*_Ca_ that has the configuration required for an effective trigger for CICR, while the slower inactivating *I*_Ca_ at the surface membrane has the configuration required to load the SR with Ca^2+^ for subsequent release [5]. The greater CDI of I_Ca_ at the t-tubules may also mean that they are an important site for Ca^2+^ autoregulation (see Section 1). Thus, effective coupling of CDI of *I*_Ca_ to SR Ca^2+^ release may result in an effective trigger for CICR and effective regulation of cell Ca^2+^ balance, controlled by SR Ca^2+^ release; conversely, extradyadic *I*_Ca_ may inactivate more slowly, thus loading the SR with Ca^2+^, suggesting that this Ca^2+^ may access extradyadic SERCA, and would play less of a role in autoregulation, as a result of being less effectively modulated by SR Ca^2+^ release.
